# AI-Guided Chemotherapy Optimization in Lung Cancer Using Genomic and Survival Data

**DOI:** 10.3390/jpm15060218

**Published:** 2025-05-27

**Authors:** Hojin Moon, Phan N. Nguyen, Jaehee Park, Minho Lee, Sohyul Ahn

**Affiliations:** 1 Department of Mathematics and Statistics, California State University, Long Beach 1250 Bellflower Blvd., Long Beach, CA 90840, USA; phan.nguyen01@student.csulb.edu; 2 Portola High School, Irvine, CA 92618, USA; 3 Department of Computer Science, Donald Bren School of Information and Computer Sciences, University of California, Irvine, CA 92697, USA; 4 Northwood High School, Irvine, CA 92620, USA

**Keywords:** artificial intelligence in oncology, genomic biomarkers, chemotherapy optimization, non-small cell lung cancer (NSCLC), survival analysis

## Abstract

**Background**: Adjuvant chemotherapy (ACT) can improve survival outcomes for patients with early-stage non-small cell lung cancer (NSCLC), but its benefit varies significantly across individuals. Identifying patients who are likely to benefit from ACT remains a critical challenge in precision oncology. **Methods**: We constructed a meta-database from two publicly available NSCLC gene expression datasets (GSE37745 and GSE29013) to address population heterogeneity. Feature selection was performed using Cox-based univariate screening with leave-one-out cross-validation. We then developed and compared three survival modeling frameworks: bagging with elastic net penalized Cox regression, Random Survival Forests (RSF), and DeepSurv neural survival networks. All models incorporated clinical covariates and selected genomic features to predict survival and recommend ACT versus observation (OBS). **Results**: Across 155 patients, RSF achieved the highest predictive performance, with a test concordance index (C-index) of0.885. Model-based recommendations were associated with improved survival in both training and test datasets, as confirmed by Kaplan–Meier analysis. Key genomic features identified included TTR, MTURN, and ETV3, suggesting their potential relevance in treatment response stratification. DeepSurv demonstrated strong predictive accuracy (C-index = 0.982) but less distinct survival curve separation compared to RSF. **Conclusions**: Our findings demonstrate that machine learning-driven survival models, particularly RSF, can effectively identify NSCLC patients who may benefit from ACT. This approach supports data-driven, individualized chemotherapy decision-making and contributes to advancing personalized treatment strategies in early-stage NSCLC.

## 1. Introduction

Lung cancer remains the leading cause of cancer-related mortality worldwide, posing a significant health challenge. In 2018, the Global Cancer Observatory (GLOBOCAN) reported approximately 2.09 million new cases of lung cancer, making it the most frequently diagnosed malignancy [1]. In the United States, lung cancer accounts for 12.7% of all cancer diagnoses, with an estimated 229,000 new cases reported in 2020 [2]. Among lung cancer subtypes, non-small cell lung cancer (NSCLC) constitutes nearly 85% of cases, encompassing adenocarcinoma, squamous cell carcinoma, and large cell carcinoma. Despite advancements in screening and treatment, long-term survival remains poor due to high recurrence rates and limited therapeutic efficacy in specific patient subgroups [3].

For patients with Stage I and II resectable NSCLC, surgical resection remains the primary curative approach. However, adjuvant chemotherapy (ACT) is commonly recommended for Stage II and select Stage IB-III cases, particularly in patients exhibiting high-risk features such as lymph node involvement, large tumor size, or specific molecular markers [4]. While ACT has demonstrated a survival benefit ranging from 4% to 15% in Stage IB-IIIA NSCLC, significant interpatient variability exists in chemotherapy response [5]. Some patients experience substantial survival benefits, while others suffer from severe toxicities with minimal therapeutic gain. This variability implies the urgent need for precision medicine strategies that accurately identify patients most likely to benefit from chemotherapy, thereby minimizing unnecessary exposure to toxicity.

Advancements in genomic profiling have revolutionized our understanding of the molecular landscape of NSCLC and its impact on chemotherapy response [6,7]. Traditional clinical parameters such as tumor size, nodal status, and histology fail to fully capture the biological heterogeneity of NSCLC. In contrast, genomic biomarkers provide a deeper understanding of tumor-specific molecular alterations that drive disease progression and therapeutic response. The integration of genomic data into clinical decision-making represents a cornerstone of precision oncology, enabling personalized treatment approaches based on tumor-specific gene expression patterns.

Genomic analysis identifies predictive biomarkers capable of distinguishing chemotherapy-responsive subgroups from those unlikely to benefit from ACT. This precision medicine framework allows treatment to be customized to each patient’s unique molecular profile, improving therapeutic efficacy while reducing unnecessary toxicity. Furthermore, genomic profiling has paved the way for targeted therapies that directly inhibit oncogenic drivers, transforming the treatment landscape for NSCLC. Despite the growing body of evidence supporting the utility of genomic biomarkers, a standardized framework for integrating genomic and clinical data into chemotherapy decision-making remains elusive [6,7].

Artificial intelligence (AI) and machine learning (ML) offer transformative potential for optimizing ACT selection in NSCLC [8,9]. AI-driven algorithms can process large-scale genomic datasets, uncover complex molecular and clinical interactions, and develop predictive models that refine chemotherapy recommendations [10]. AI-based methods outperform conventional statistics by capturing nonlinear patterns, identifying subtle genomic signals, and improving patient stratification [11].

Recent advanced ML models have demonstrated superior predictive performance compared to traditional survival models, particularly in genomic data contexts, where feature selection and dimensionality reduction are critical [12]. AI-driven survival analysis further enables subgroup identification, revealing distinct patient clusters with varying survival trajectories based on genomic signatures [13]. These insights inform more precise treatment decisions, ensuring that ACT is selectively administered to patients with a high likelihood of benefit [14].

Moon et al. [15,16,17,18] developed statistical decision support tools to identify key risk factors and estimate the likelihood of benefit from ACT in early-stage NSCLC patients. Their earlier studies used a single gene expression dataset from a randomized clinical trial to develop models that identified genomic markers predictive of treatment response, allowing stratification between patients likely to benefit from ACT and those for whom surgical resection alone (OBS) may be sufficient. Collectively, these studies demonstrate the potential of genomics-informed ACT selection to improve treatment decision-making in NSCLC. Further validation with external cohorts and larger sample sizes will be important to strengthen predictive reliability and clinical relevance.

Our study represents a significant step forward in clinical decision support for lung cancer treatment. By integrating advanced machine learning algorithms—including a bagging approach based on the penalized Cox Proportional Hazards (PH) models [19,20], the Random Survival Forests (RSF) [21], and a deep learning survival network (DeepSurv) [22]—we aim to enhance the robustness and accuracy of AI-driven treatment recommendations. These sophisticated algorithms enable more precise survival predictions while providing therapeutic strategies for individual patient profiles. By optimizing chemotherapy selection, this approach has the potential to improve clinical outcomes, minimize unnecessary toxicity, and advance precision oncology in NSCLC.

Despite these advances, further refinement of AI-based predictive models is necessary to enhance their clinical applicability. The integration of diverse multi-omics datasets, incorporation of clinical data, and external validation of predictive models in independent patient cohorts are crucial next steps. In this paper, we demonstrate how AI-driven survival analysis and genomic profiling enhance personalized chemotherapy decision-making, ultimately leading to improved clinical outcomes for early-stage NSCLC patients.

## 2. Materials and Methods

### 2.1. Data Description

Identifying reliable gene signatures for lung cancer remains a significant challenge due to the complexity and heterogeneity of genomic data. Traditional approaches relying on a single dataset often lack reproducibility and consistency across independent studies. To address these limitations, we have adopted a meta-database approach that aggregates and synthesizes data from two independent publicly available gene expression datasets (GSE37745 and GSE29013). This method enhances the robustness and generalizability of findings by mitigating dataset-specific variability and improving statistical power. By integrating data from multiple sources, we aim to establish a more comprehensive and accurate understanding of the molecular mechanisms underlying lung cancer progression and treatment response.

The gene expression datasets GSE37745 and GSE29013 were selected for this study based on their relevance to early-stage NSCLC and the availability of key clinical annotations, including adjuvant chemotherapy status, age, sex, and clinical stage. Both datasets used the Affymetrix Human Genome U133 Plus 2.0 microarray platform, enabling direct probe-level integration without requiring cross-platform harmonization.

To ensure data comparability and reduce batch effects, we applied the Robust Multi-array Average (RMA) method for background correction, normalization, and summarization of probe intensities within each dataset. After merging, we further performed quantile normalization and median centering across all samples to harmonize expression values. Only patients with consistent clinical annotations and stage IB–II NSCLC were included in the final cohort. While we could not completely rule out residual batch effects, our preprocessing pipeline aimed to minimize their impact and ensure a unified analysis across datasets.

This study utilized two publicly available datasets from the NCBI Gene Expression Omnibus (GEO) repository. The first dataset, GSE37745 [23], comprised gene expression profiles from 196 NSCLC patients treated between 1995 and 2005. This dataset included tumor and non-tumor tissue samples analyzed using Affymetrix Human Genome U133 Plus 2.0 Array, enabling a comparative study of cancer-specific gene expression patterns. Additionally, the dataset incorporated clinical information obtained from a regional lung cancer registry, providing long-term follow-up data. Of the 196 patients, 71 underwent OBS, while 29 received ACT. The remaining 96 patients lacked detailed treatment information; thus, our analysis focused on 100 patients with complete therapeutic records.

The second dataset, GSE29013 [24], consisted of gene expression profiles from 55 NSCLC patients. These samples were analyzed using Affymetrix U133 Plus 2.0 arrays, recognized for their high sensitivity and broad gene coverage. The dataset was divided into two treatment groups: 21 patients underwent OBS, receiving only routine monitoring without active intervention, while 34 patients received ACT. This structured dataset enabled a comparative analysis of gene expression differences between treatment groups, facilitating the identification of potential biomarkers associated with chemotherapy response.

By utilizing these datasets, our study aims to improve the identification of predictive gene signatures that guide ACT selection in NSCLC. The combination of multiple independent datasets enhances model robustness, strengthens statistical validity, and reduces the risk of overfitting. Ultimately, our data-driven approach supports the development of precision medicine strategies, improving treatment efficacy and minimizing unnecessary chemotherapy exposure.

### 2.2. Model Design

The datasets comprising 155 patients were divided into training and testing subsets to ensure a rigorous evaluation of predictive performance. Specifically, 80% of the data were allocated for training, while the remaining 20% were designated for model testing. In GSE29013, the training set included 44 patients, with 11 patients reserved for testing. Similarly, in GSE37745, 80 patients were used for training, while 20 patients constituted the test set. The treatment distribution varied between datasets: GSE29013 included 34 ACT and 21 OBS patients, whereas GSE37745 consisted of 29 ACT and 71 OBS patients. A summary of patient demographics for both datasets, stratified by training and testing set, is presented in Table 1.

Each dataset contained 54,675 probe sets, requiring a rigorous feature selection process to extract the most relevant predictors of patient survival. A systematic screening process was employed to identify informative probe sets while minimizing noise that could compromise model performance. This step was essential to refining the predictive framework and improving survival outcome estimations.

**Figure 1:**
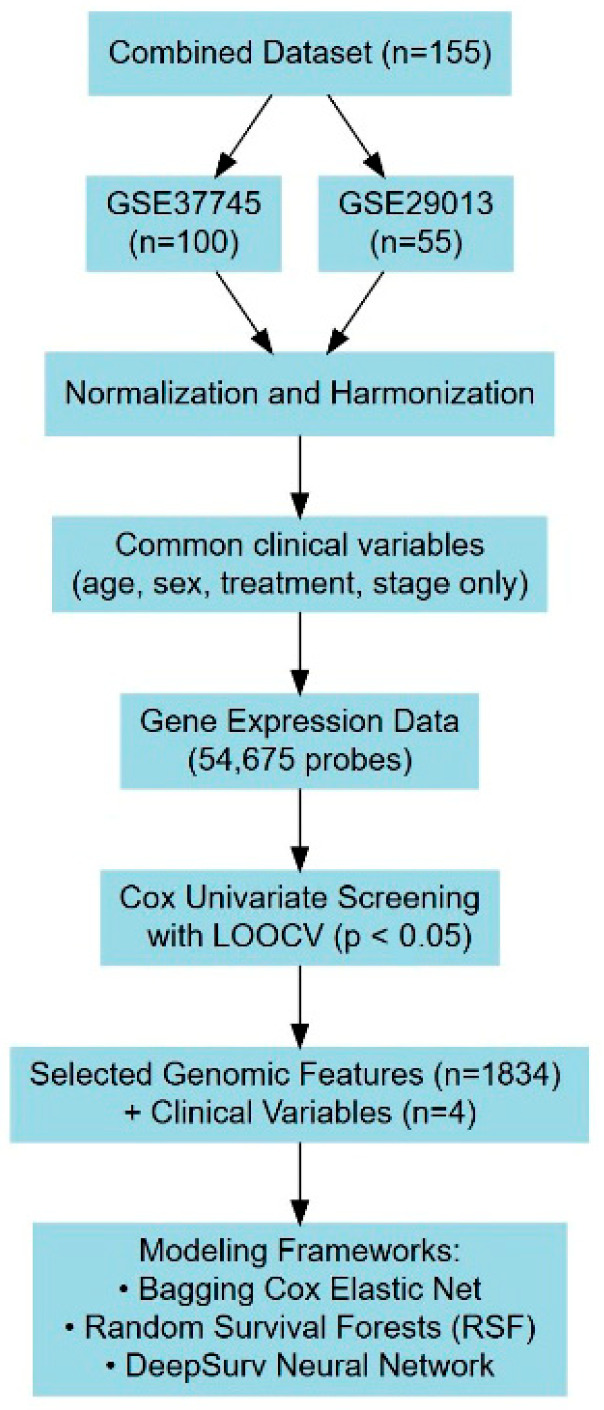
A schematic diagram summarizing model design workflow.

To optimize model performance, an initial leave-one-out cross-validation (LOOCV) procedure was conducted alongside univariate analysis using Cox PH models [25]. Probe sets were selected based on a 5% significance threshold, ensuring that only statistically meaningful variables were retained. Each probe was assigned a variable importance score based on the frequency with which it was identified as significant. The training dataset was subsequently used to develop predictive models incorporating both genomic features and key clinical and demographic covariates, including age, sex, and clinical stage. These refinements aimed to maximize predictive accuracy and establish a robust framework for ACT benefit estimation in NSCLC patients. A schematic diagram in Figure 1 summarizes the LOOCV and model design workflow.

#### 2.2.1. Regularized Cox Proportional Hazards Model

The Cox PH model [25] is a widely used statistical method in survival analysis, allowing for the examination of multiple covariates on time-to-event outcomes such as mortality or disease recurrence. A key assumption of this model is proportionality of hazards, which implies that the relative risk between individuals remains constant over time.

In this study, survival data are represented as $\left(t_{i},\;\delta_{i},\;Z_{i}\right),\;$ where $t_{i}$ is the observed survival or censoring time, $\delta_{i}$, indicates the censor status (1 for event, 0 for censored), and $Z_{i}=\left(Z_{i1},\;Z_{i2},\ldots ,Z_{ip}\right)$ represents a vector of covariates for the *i*-th individual. The Cox PH model is expressed as:

$$h\left(t|Z_{i}\right)=h_{0}\left(t\right)\exp\left(\overset{p}{\underset{j=1}{\sum }}\beta_{j}Z_{ij}\right),$$

where $h_{0}\left(t\right)$ is the baseline hazard function, and $\beta_{j}$ are the regression coefficients estimated by maximizing the partial likelihood:

(1) $$L_{p}\left(\beta \right)=\overset{n}{\underset{i=1}{\prod }}{\left\{\frac{\exp\left(\sum_{j=1}^{p}\beta_{i}Z_{i}\right)}{\sum_{j\in R\left(t_{i}\right)}\exp\left(\sum_{k=1}^{p}\beta_{k}Z_{jk}\right)}\right\}}^{\delta_{i}},$$

where $R\left(t_{i}\right)$ represents the set of individuals at risk at time $t_{i}.$

The hazard ratio (HR) quantifies the relative risk of an event occurring under different treatment conditions. For treatment groups $Z=Z_{1}$ (e.g., OBS) and $Z=Z_{2}$ (e.g., ACT), the HR is defined as:

(2) $$HR=\frac{h\left(t|Z=Z_{1}\right)}{h\left(t|Z=Z_{2}\right)}=\exp\left(\overset{p}{\underset{j=1}{\sum }}\beta_{i}\left(Z_{1j}-Z_{2j}\right)\right),$$

where β represents the estimated model coefficients, and the exponent represents the difference in risk based on their respective treatments.

Given the nature of genomic data, traditional Cox regression models often suffer from overfitting and high variance, leading to unreliable predictions. To address these challenges, regularized Cox regression with an elastic net penalty [20] is employed. The elastic net penalty combines $L_{1}$ (lasso) and $L_{2}$ (ridge) regularization, enhancing variable selection while maintaining model stability. The penalized function is defined as:

$$penalty=\lambda \left(\alpha \overset{p}{\underset{j=1}{\sum }}\left|\beta_{j}\right|+\frac{1-\alpha }{2}\overset{p}{\underset{j=1}{\sum }}\beta_{j}^{2}\right),$$

where λ is the tuning parameter controlling the overall strength of regularization, and α is the mixing parameter balancing the $L_{1}$ (lasso, α=1) and $L_{2}$ (ridge, α=0) penalties.

The objective function for elastic net-regularized Cox regression is given by:

$$L\left(\beta \right)=\frac{1}{n}\overset{n}{\underset{i=1}{\sum }}\delta_{i}\left(Z_{i}^{T}\beta -\log\underset{j\in R_{i}}{\sum }e^{Z_{j}^{T}\beta }\right)-penalty,$$

where $L\left(\beta \right)$ is the penalized partial log-likelihood, $Z_{i}^{T}\beta$ is the linear predictor for the i-th individual, $R_{i}$ is the risk set at time $t_{i}$, and $\delta_{i}$ is an event indicator (1 for event, 0 for censored).

The hyperparameters α and λ were optimized through a systematic grid search during the training phase. A sequence of 101 candidate values for α (ranging from 0 to 1 in increments of0.01) was evaluated, with λ determined via LOOCV for each α. The optimal α-λ combination yielding the best predictive performance was selected.

To further enhance model stability and predictive accuracy, bagging (bootstrap aggregating) [19] was incorporated. This ensemble learning algorithm reduced variance by averaging predictions from multiple bootstrapped Cox models. Each bootstrap sample was drawn with replacement from the training data, and a regularized Cox PH model was trained on each resampled dataset. The final ensemble model aggregated individual model predictions to generate a robust estimate of the survival risk.

Patient treatment recommendations are based on the predicted hazard ratio (HR), ensuring an evidence-based, individualized decision-making approach. Specifically, HR > 1 suggests that the hazard associated with $Z_{1}$ (e.g., OBS) exceeds that of $Z_{2}$ (e.g., ACT), favoring ACT. Conversely, HR < 1 indicates that the hazard under OBS is lower, supporting a recommendation for OBS.

The model was implemented in R using the *glmnet* package [26] for elastic net regularization, and the bagging process was conducted through repeated bootstrapping in R.

#### 2.2.2. Random Survival Forests Model

Random Survival Forests (RSF [21]) is a nonparametric ensemble learning algorithm specifically designed for survival analysis, extending the principles of random forests [19] to accommodate time-to-event data. RSF employs bootstrap aggregation (bagging) and recursive partitioning to construct an ensemble of survival trees from randomly selected subsets of predictors. Unlike conventional Cox PH models, RSF does not impose proportional hazards assumption and effectively captures complex relationships among covariates.

For a dataset with n individuals, a bootstrap sample is generated by randomly drawing n individuals with replacement from the original dataset: $D=\left\{\left(x_{1},t_{1},\delta_{1}\right),\ldots \left(x_{n},\;t_{n},\;\delta_{n}\right)\right\},$ where $x_{i}$ represents the covariates for individual i, $t_{i}$ denotes the follow-up time, and $\delta_{i}$ is the censoring indicator. Each tree m is trained using a bootstrap sample $D^{\left(m\right)}$, with $\left|D^{\left(m\right)}\right|=n.$ Individuals excluded from a given bootstrap sample form an out-of-bag (OOB) set, which serves as an internal validation set for assessing prediction accuracy.

Each tree is grown via recursive partitioning, wherein at each node, a random subset of predictors is selected, and candidate split points are evaluated to maximize survival differentiation between resultant child nodes. The log-rank statistic [27] is utilized as the splitting criterion to partition nodes in a manner that optimally stratifies survival outcomes. The tree-building process continues until a predefined minimum terminal node size is reached or when no further improvement in survival separation is possible.

The RSF model estimates the relationship between covariates $Z_{i}$ and survival outcomes by aggregating survival predictions across multiple trees. Given an ensemble of M survival trees, the cumulative hazard function (CHF) for an individual i under treatment $Z_{\left(i,k\right)}$ (where $Z_{\left(i,1\right)}$ denotes OBS and $Z_{\left(i,2\right)}$ denotes ACT) is obtained by averaging the CHFs from all trees:

$${\hat{H}}_{RSF,i}^{\left(Z_{\left(i,k\right)}\right)}\left(t\right)=\frac{1}{M}\overset{M}{\underset{m=1}{\sum }}{\hat{H}}_{i,m}^{\left(Z_{\left(i,k\right)}\right)}\left(t\right),$$

where ${\hat{H}}_{i,m}^{\left(Z_{\left(i,k\right)}\right)}\left(t\right)$ is the CHF predicted by tree m for treatment $Z_{\left(i,k\right)}$, and M represents the total number of trees in the forest. Using this aggregated hazard function, the individualized survival probability of patient i under treatment $Z_{\left(i,k\right)}$ at time t is computed as:

$${\hat{S}}_{RSF,i}^{\left(Z_{\left(i,k\right)}\right)}\left(t\right)=\underset{u\leq t}{\prod }\left(1-{\hat{H}}_{RSF,i}^{\left(Z_{\left(i,k\right)}\right)}\left(u\right)\right),$$

where ${\hat{H}}_{RSF,i}^{\left(Z_{\left(i,k\right)}\right)}\left(u\right)$ represents the CHF at time u.

RSF offers several advantages for personalized survival modeling in clinical decision-making. It is especially effective at handling very large numbers of features while reducing the risk of overfitting through ensemble averaging. One of RSF’s salient features is its ability to quantify variable importance, which provides insight into the relative contribution of each predictor. Variable importance is assessed by permuting the values of a given predictor in the OOB samples and measuring the resultant decline in prediction accuracy. Variables with higher importance scores exert greater influence on survival outcomes.

For individualized treatment recommendations, survival probabilities ${\hat{S}}_{RSF,i}^{\left(Z_{\left(i,1\right)}\right)}\left(t\right)$ and ${\hat{S}}_{RSF,i}^{\left(Z_{\left(i,2\right)}\right)}\left(t\right)$ are compared over multiple time horizons. Treatment selection is determined based on the following decision rule: If ${\hat{S}}_{RSF,i}^{\left(Z_{i,1}\right)}\left(t\right)>{\hat{S}}_{RSF,i}^{\left(Z_{i,2}\right)}\left(t\right),$ OBS is recommended. Otherwise, ACT is recommended. The treatment associated with the higher survival probability is deemed preferable.

The RSF model was implemented using the *randomForestSRC* package in R [28]. Hyperparameters—including the number of trees, the random split points, the number of variables randomly selected for node splitting, and the minimum terminal node size—were optimized via LOOCV to enhance model robustness and generalizability.

#### 2.2.3. Deep Learning Survival Model

DeepSurv [22] is an advanced deep neural network-based survival model that extends the Cox Proportional Hazards framework by capturing intricate nonlinear relationships and complex interactions among covariates. Unlike the standard Cox model, which assumes a linear log-hazard function, DeepSurv leverages the representational power of deep learning to enhance individualized survival prediction and treatment effect estimation. The model formulates the hazard function for an individual i as:

$$h\left(t|x_{i}\right)=h_{0}\left(t\right)\exp\left(\hat{f}\left(x_{i};\theta \right)\right),$$

where $h_{0}\left(t\right)$ represents the baseline hazard function, and $\hat{f}\left(x_{i};\theta \right)$ denotes the log hazard ratio, predicted by a deep neural network parameterized by θ.

**Figure 2:**
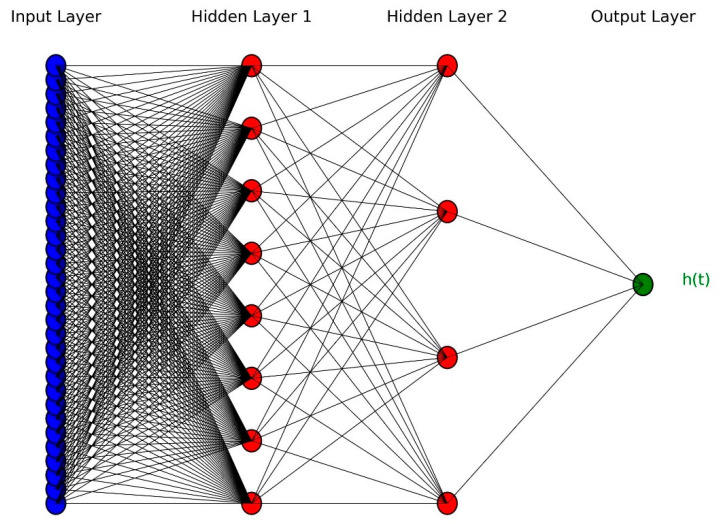
An example of DeepSurv architecture with two hidden layers.

DeepSurv employs a feedforward neural network (FNN) to estimate the log hazard ratio $f\left(x_{i};\theta \right)$ using an individual’s covariate vector $x_{i}$ as input. The architecture consists of an input layer that encodes patient-specific features, multiple fully connected hidden layers with nonlinear activation functions to model complex feature interactions, and a single output neuron without an activation function that directly predicts the log hazard ratio. The model architecture is structured to capture the complex interplay of patient characteristics and survival outcomes, making it a powerful tool for personalized risk assessment. Figure 2 illustrates an example DeepSurv network, comprising 32 input units, two hidden layers with eight and four neurons, respectively, and a single output layer.

The loss function in DeepSurv is derived from the negative partial likelihood of the Cox model, as introduced in Equation (1) in Section 2.2.1. DeepSurv optimizes the negative partial log-likelihood function:

$$l\left(\theta \right)=\underset{i\in D}{\sum }\left(\hat{f}\left(x_{i};\theta \right)-\log\underset{j\in R_{i}}{\sum }\exp(\hat{f}\left(x_{i};\theta \right))\right),$$

where D is a set of individuals who experienced the event, and $R_{i}$ denotes the individuals still at risk at time $T_{i}$. To mitigate overfitting and enhance generalization, an elastic net regularization term $R\left(\theta \right)$ is incorporated into the loss function: $R\left(\theta \right)=\lambda_{1}{||\theta ||}_{1}+\lambda_{2}{||\theta ||}_{2}^{2},$ where $\lambda_{1}$ induces sparsity, and $\lambda_{2}$ controls overfitting by penalizing large coefficients. The final loss function integrates both terms: $L_{f}\left(\theta \right)=-l\left(\theta \right)+R\left(\theta \right).$

The loss function is minimized using an optimization algorithm such as stochastic gradient descent (SGD) algorithm with backpropagation, iteratively updating parameters to minimize the loss:

$$\theta_{updated}=\theta_{old}-\eta \frac{\partial L_{f}\left(\theta \right)}{\partial \theta },$$

where η represents the learning rate. The model undergoes training on a dedicated training dataset, with hyperparameters such as the learning rate, regularization terms, hidden layers, dropout rates, and activation functions tuned based on validation performance.

To efficiently navigate the hyperparameter space, Bayesian Hyperparameter Optimization (BHO) [29] was employed. Unlike grid search or random search, which evaluate hyperparameters through exhaustive or arbitrary sampling, BHO leveraged probabilistic models to identify optimal configurations efficiently. By systematically exploring hyperparameter interactions, BHO reduced computational overhead while improving model generalization. This method was particularly beneficial when working with large numbers of variables, where traditional tuning methods may have been inefficient.

The architecture of a deep neural network is primarily defined by its hidden layers, both in terms of the number of layers and the number of neurons per layer. To enhance generalization and prevent overfitting, dropout regularization is applied during training, where certain neurons are randomly deactivated in each iteration. This technique reduces dependency on specific neurons and forces the model to learn more robust representations. In addition to dropout, batch normalization (BN) is implemented to stabilize training and accelerate convergence by standardizing activations across intermediate layers of the network [30]. Another enhancement involves the use of scaled exponential linear units (SELU) as activation functions, which autonomously normalize network activations, thereby improving training stability and convergence rates [31]. The model is implemented in Python (version 3.12.4) using TensorFlow/Keras model architecture.

Using DeepSurv, treatment recommendations are generated based on the logarithmic transformation of the hazard ratio in Equation (2). For each patient, the algorithm estimates risk across different treatment options, assuming a common baseline risk level. By comparing the log hazard values under different treatments, DeepSurv identifies the optimal course of action. The risk differential between treatment strategies is quantified as the recommender function:

$$rec_{ij}\left(x\right)=\log\left(HR\right)={\hat{h}}_{i}\left(x\right)-{\hat{h}}_{j}\left(x\right),$$

where ${\hat{h}}_{i}\left(x\right)$ and ${\hat{h}}_{j}\left(x\right)$ denote the predicted log hazards for treatment i (e.g., OBS) and j (e.g., ACT), respectively. A positive $rec_{ij}\left(x\right)$ value suggests that treatment i (OBS) is associated with a higher risk than treatment j (ACT), leading to a recommendation for ACT. Conversely, a negative $rec_{ij}\left(x\right)$ implies that treatment i (OBS) is more favorable, as it is associated with a lower risk compared to treatment j (ACT).

By incorporating deep learning techniques into survival analysis, DeepSurv enhances personalized treatment recommendations, allowing for more precise, risk-adjusted decision-making in clinical settings. The ability to model complex interactions and individualized hazard ratios makes DeepSurv a powerful alternative to traditional survival models, particularly in complex datasets with large numbers of covariates.

#### 2.2.4. Performance Measure: Concordance Index

The concordance index (C-index) was employed to assess the predictive efficacy of the survival models [32]. Widely recognized in survival analysis, the C-index quantifies the discriminative capacity of a prognostic model by evaluating its ability to correctly rank survival times. Specifically, it determines whether individuals with higher predicted risk scores encounter the event of interest (e.g., mortality) earlier than those with lower risk estimates. As a fundamental criterion in prognostic modeling, the C-index provides an objective measure of predictive accuracy, thereby ensuring the reliability of individualized treatment recommendations [33].

The C-index is defined as follows:

$$C=\frac{\sum_{\left\{i,j\right\}\in C}I\left(h\left(X_{i}\right)>h\left(X_{j}\right)\right)}{\left|C\right|},$$

where C denotes the set of all permissible (comparable) patient pairs $\left\{i,j\right\}$ satisfying $T_{i}<T_{j}$, meaning that patient i experiences an event prior to patient j. The function $h\left(X\right)$ represents the predicted risk score or hazard function derived from the survival model. The indicator function $I\left(\cdot \right)$ assumes a value of 1 if the predictive risk satisfies $h\left(X_{i}\right)>h\left(X_{j}\right)$, and 0 otherwise. The denominator $\left|C\right|$ represents the total number of comparable patient pairs.

A C-index of 0.5 suggests that the model’s predictive performance is equivalent to random choice, whereas a C-index of 1.0 indicates perfect concordance, signifying that the model ranks all survival times correctly [34]. In practical applications, a C-index exceeding0.7 is generally considered indicative of favorable discriminative power [35], although values above0.8 are preferable for robust predictive performance.

Given the prevalence of right-censored survival data, Harrell’s C-index is commonly employed, as it accounts for censored cases by excluding non-informative pairs in which the event time remains unobserved [36]. This adaptation enhances the robustness of model evaluation, ensuring reliable performance assessments even in datasets with substantial censoring.

In the realm of personalized medicine for NSCLC, the C-index serves as a critical benchmark for evaluating survival models. Its capacity to assess predictive discrimination is integral to the development of evidence-based, individualized treatment strategies, thereby reinforcing its significance in clinical decision-making frameworks.

## 3. Results

To evaluate the predictive performance of the survival models, the C-index was computed on the test dataset for three distinct methodologies: bagging with regularized Cox regression, RSF, and DeepSurv. This section systematically examined their effectiveness in providing patient-specific survival predictions and treatment recommendations.

For feature screening and preprocessing, a treatment interaction Cox regression model was employed, identifying relevant predictors associated with patient outcomes. LOOCV was applied to the training dataset (summarized in Table 1), wherein probe sets with p>0.05 were excluded from further analysis. This process reduced the initial pool of 54,675 probe sets to a refined subset of 1834, in addition to four clinical and demographic variables—age, sex, treatment, and stage. These screened variables were consistently utilized across all three survival models to maintain a generalized comparison framework.

The bagging with a regularized Cox model was trained using 200 bootstrap resamples, estimating risk scores that informed treatment recommendations. Within the training dataset, the model assigned 82 patients to the OBS category and 42 patients to the ACT category. Among these, 112 patients adhered to the model’s recommendation (either ACT or OBS), while 12 did not. Similarly, within the test dataset, the model classified 29 patients as OBS and 2 patients as ACT, of whom 16 adhered to the recommendation and 15 did not.

**Figure 3:**
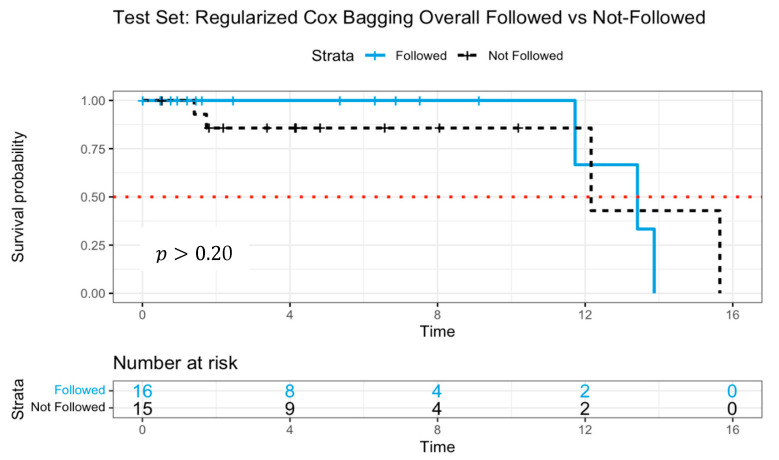
Survival curves of patients following versus not following model recommendations using Cox proportional hazards with log-rank test. The dotted line indicates median survival.

The C-index for this model was0.996 in the training dataset, demonstrating near-perfect discrimination, while in the test dataset, the C-index declined to 0.709, indicating moderate predictive performance. The Kaplan–Meier survival curve [37], illustrated in Figure 3, depicted the survival probabilities of patients who followed versus did not follow the model’s treatment recommendation. Patients adhering to the model’s guidance exhibited a higher survival probability over approximately 11 years, although mortality events were observed in both groups between 12 and 16 years. The estimated median survival times were 13.4 years for patients who followed and 12.2 years for patients who did not. While there was a visible difference in survival probability up until around the 11-year mark, the log-rank test comparing survival probabilities between the groups yielded p>0.20, suggesting insufficient statistical evidence to assert a significant distinction in survival distributions.

The absence of statistical significance, despite the observed survival advantage, may be attributed to the limited sample size, which likely constrained the statistical power needed to detect meaningful differences. Notably, by year 12, only two patients remained at risk in the group that adhered to model recommendations. Additionally, the model’s recommendations may not provide sufficient separation in survival probabilities, indicating potential avenues for further refined models.

To further enhance predictive accuracy from the bagging approach with regularized Cox regression models, the RSF was employed with the same training and test data in Table 1. Hyperparameter tuning was conducted via 10-fold cross-validation, optimizing key parameters, including the total trees in the forest (*ntree* = 1000), the number of variables considered per split (*mtry* = 37), the minimum node size for splitting (*nodesize* = 6), the number of potential splits (*nsplit* = 10), and the splitting criterion as a log-rank test.

Following RSF-based treatment recommendations, the model classified 83 patients as OBS and 41 patients as ACT in the training dataset. Of these, 75 patients complied with the model’s recommendation, while 49 deviated. In the test dataset, 20 patients were recommended for OBS, whereas 11 were assigned to ACT. Among them, 17 followed the model’s suggestion, while 14 did not.

The mean C-index for RSF was0.889 in the training dataset and0.885 in the test dataset, reflecting strong discriminative ability. Table 2 presents variable importance scores, quantifying the relative influence of each predictor in survival outcome predictions. Notably, transthyretin (TTR) exhibited the highest importance score, implicating its role in neurodegenerative diseases and systemic amyloidosis. Other genes, such as PREPL (prolyl endopeptidase-like—usually involved in neurological and metabolic pathways) and MTURN (a neural progenitor differentiation regulator associated with cancer progression), emerged as potential biomarkers influencing survival outcomes.

**Figure 4:**
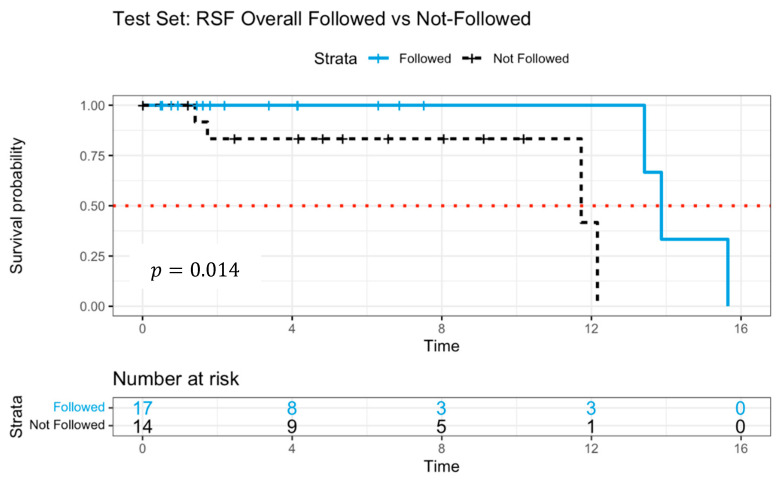
Survival curves of patients following versus not following model recommendations using Random Survival Forests with log-rank test.

Figure 4 illustrates the Kaplan–Meier survival curves, distinguishing patients who adhered to versus those who deviated from RSF model recommendations. The survival probability was significantly higher among patients who followed the RSF guidance (p=0.014), reinforcing the model’s predictive efficacy. The median survival time for the patients who followed was 13.9 years, whereas non-adherent patients exhibited 11.7 years, a steeper decline in survival probability, suggesting poorer prognosis.

The RSF findings indicated that adhering to model-recommended treatment strategies was associated with enhanced survival outcomes. To further validate these results, a deep learning survival model (DeepSurv) was implemented as an additional comparative framework.

Our third model, the DeepSurv neural network, was trained with the same dataset, optimizing hyperparameters as follows: learning rate = 0.3, dropout = 0.5, learning rate decay = 1.0, L2 regularization = 2.46, L1 regularization = 4.14, batch normalization = true, and standardization of input features = true. Network architecture consisted of two hidden layers (100 and 50 neurons), and SELU activation function. The resulting survival neural network comprised 1838 input neurons, two hidden layers, and a single output layer.

DeepSurv classified 1 patient as OBS and 92 patients as ACT in the training dataset, where 56 followed the recommendations and 37 did not. In the test dataset, 2 patients were assigned to OBS and 29 to ACT, with 16 adhering to the model’s guidance and 15 diverging.

**Figure 5:**
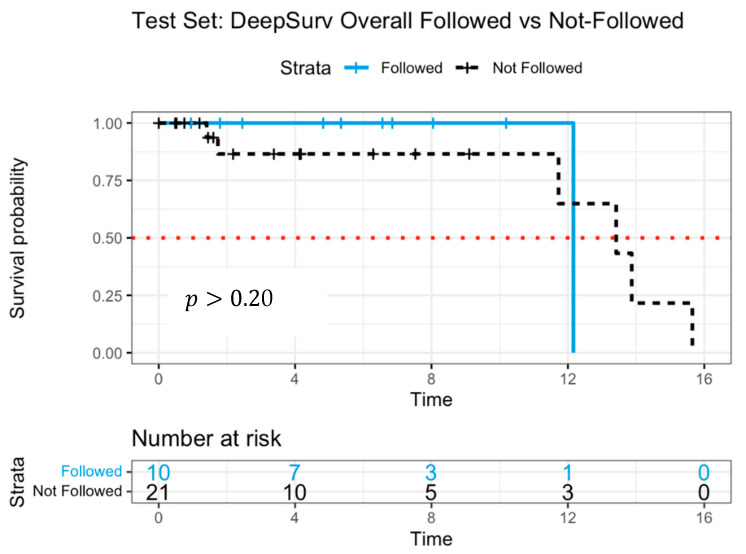
Survival curves of patients following versus not following model recommendations using DeepSurv with log-rank test.

The C-index for DeepSurv was 0.990 in the training data and 0.982 in the test data, indicating high predictive accuracy. Figure 5 presents Kaplan–Meier survival curves, comparing adherent and non-adherent groups. Initially, the “followed” group (solid line) demonstrated slightly better survival probabilities than the “not-followed” group (dashed line) for up to 12 years. However, the survival trajectories suggested a less distinct separation compared to RSF, indicating a more limited predictive distinction. The median survival time was estimated at 12.2 years for the followed group and 13.4 years for the not-followed group.

The lack of significant survival differentiation in the DeepSurv model may be attributed to the limited number of patients at later time points, which could affect the reliability of survival estimates. While the overall survival trend aligns with RSF findings, the smaller separation between survival curves suggests that RSF may offer a more robust differentiation of treatment effects.

To provide a clearer comparison of the modeling approaches, we summarized the key features and performance metrics of the three models used in this study in Table 3. While all three models utilized the same feature set selected via LOOCV-based Cox screening, they differed in their handling of feature interactions, assumptions, and predictive behavior. Notably, RSF achieved the most consistent discriminative performance across both training and test datasets, while DeepSurv demonstrated the highest C-index but with less separation in survival curves. The Bagging Cox model showed strong performance in the training set but declined in generalizability, likely due to limitations in modeling complexity and interactions.

## 4. Discussion

This study presents an advanced clinical decision support framework for optimizing adjuvant chemotherapy (ACT) decisions in non-small cell lung cancer (NSCLC) patients. By integrating genomic data with sophisticated machine learning models—including bagging with regularized Cox regression, Random Survival Forests (RSF), and Deep Survival Networks (DeepSurv)—we have developed a predictive system designed to enhance personalized treatment strategies. The results demonstrate that machine learning-driven survival analysis effectively identifies patient subgroups most likely to benefit from ACT, thereby reducing unnecessary chemotherapy exposure while improving survival outcomes.

Several prior studies have explored machine learning techniques for survival prediction and treatment response in NSCLC. For example, Huang et al. [13] demonstrated the predictive potential of support vector machines on NSCLC genomic data, reporting a C-index of0.84, although their model did not generate individualized treatment recommendations. Moon et al. [15] previously applied Cox-based models for ACT benefit prediction using a single dataset, but they lacked external validation or model comparison. Other studies have underscored the utility of ensemble learning and deep neural networks for survival analysis in cancer populations [12]. Our study builds upon these efforts by incorporating two independent datasets, applying consistent LOOCV-based feature selection, and evaluating three distinct survival modeling frameworks. This comprehensive comparison—summarized in Table 3—demonstrates the potential for RSF to achieve a favorable balance between predictive accuracy and survival curve discrimination, while also illustrating the high concordance performance of DeepSurv.

Through validation using two independent datasets from the National Center for Biotechnology Information (NCBI), our models exhibited strong predictive performance, as reflected by high concordance index values. In terms of biomarker discovery, our RSF model identified several gene features with potential relevance to chemotherapy response. We recognize the importance of contextualizing these findings. For example, transthyretin (TTR) has been proposed as a prognostic marker in NSCLC and linked to survival outcomes [39]. TTR encodes a transport protein primarily involved in the distribution of thyroid hormones and retinol. Emerging evidence suggests that low serum TTR levels are associated with poor nutritional status and increased systemic inflammation, both of which are negative prognostic indicators in cancer patients undergoing chemotherapy. Specifically, Shimura et al. [39] reported that TTR serves as a marker for predicting treatment outcomes and tolerance in lung cancer patients undergoing chemoradiotherapy.

MTURN (Maturin), a neural progenitor differentiation regulator, was recently implicated in platelet-derived mRNA signatures for lung cancer diagnosis [41]. MTURN is a neural differentiation regulator with emerging roles in cancer biology. Although not widely studied in NSCLC, MTURN has been implicated in blood-based mRNA signatures for lung cancer detection, particularly in platelet-derived RNA profiles [41]. Its expression may reflect tumor–platelet interactions, which are increasingly recognized as contributors to metastasis and treatment resistance.

ETV3 (ETS Variant Transcription Factor 3), a member of the ETS transcription factor family, has been associated with tumor suppression in lung adenocarcinoma [42]. ETV3 is a transcriptional repressor that modulates cell cycle progression, immune signaling, and interferon responses. Dysregulation of ETV3 has been linked to tumor immune evasion, potentially influencing how tumor cells respond to systemic therapies. In lung cancer, gene fusions involving ETV3 have been observed and may contribute to oncogenic transcriptional reprogramming [42]. These mechanistic insights enhance the biological interpretability of our modeling results and suggest that genes like TTR, MTURN, and ETV3 may serve not only as predictors of treatment response but also as potential biomarkers for patient stratification in future clinical studies. Those references were now summarized in Table 2 to help position our genomic findings within the broader literature. Further investigation into these biomarkers may provide deeper insight into chemotherapy response mechanisms and contribute to more refined treatment selection criteria.

Among the models evaluated, RSF achieved the most pronounced survival differentiation, suggesting its potential utility in individualized treatment guidance. While DeepSurv displayed a high C-index, its survival curve separation was less distinct, suggesting that DeepSurv may be less effective in distinguishing survival outcomes due to its model complexity. Future research should incorporate larger, more diverse datasets and explore alternative feature selection methods to improve predictive accuracy and generalizability.

Beyond optimizing chemotherapy decisions, this study illustrates the expanding role of artificial intelligence (AI) in precision oncology. AI-driven approaches enable more effective patient stratification based on genomic risk factors, ensuring that chemotherapy interventions are both targeted and clinically meaningful. These advancements hold significant implications for reducing treatment toxicity, enhancing patient quality of life, and improving healthcare resource allocation. Further developments should focus on expanding model applicability to broader patient populations and integrating additional multi-omics data, such as proteomics, transcriptomics, and metabolomics, to refine predictive performance.

This study has several limitations that warrant consideration. First, the relatively small size of the test dataset (n=31) may limit the statistical power to detect significant survival differences, particularly in subgroup analyses. Moreover, the limited demographic diversity within GSE37745 and GSE29013 may restrict the generalizability of our findings to broader patient populations. While the RSF model demonstrated strong discriminative ability and DeepSurv achieved high predictive accuracy, their generalizability remains to be validated in broader and more heterogeneous populations. Second, although we combined two publicly available datasets to improve robustness, external validation using independent cohorts—such as those from The Cancer Genome Atlas (TCGA)—is necessary to confirm the reproducibility and applicability of our findings. Lastly, although we applied a robust preprocessing pipeline—including RMA normalization, quantile normalization, and median centering—to harmonize gene expression data across GSE37745 and GSE29013, residual batch effects cannot be entirely ruled out. Future studies will focus on integrating additional cohorts with well-annotated clinical data to enhance statistical power and evaluate model performance across diverse patient populations. Additionally, harmonizing multi-omics datasets, such as proteomic or epigenetic data, may further improve the precision of treatment recommendations. These extensions will be crucial for translating the proposed modeling framework into clinically actionable tools for personalized decision-making in NSCLC.

From a clinical perspective, the models developed in this study offer a data-driven framework for optimizing ACT decisions in early-stage NSCLC. Each model is based on open-source statistical software and can be reproduced in typical academic or clinical research environments. RSF, in particular, offers a transparent view of variable importance, which supports clinician interpretation and model transparency. While DeepSurv achieves high predictive accuracy, its complexity may limit interpretability in practice unless supplemented with explainable AI tools (e.g., SHAP values). Practical implementation will require further validation, integration with electronic health record systems, and user-friendly interfaces to support oncologist decision-making.

Importantly, the ethical application of these models in clinical settings requires careful consideration. Predictive algorithms should not replace clinical judgment but rather assist in identifying patients most likely to benefit from ACT based on individualized genomic and clinical profiles. Moreover, algorithmic bias, particularly due to the underrepresentation of minority populations in training data, must be addressed through diverse cohort validation and fairness auditing.

In conclusion, this study establishes a scientifically rigorous and clinically impactful framework for data-driven chemotherapy decision-making in NSCLC. The findings provide a foundation for next-generation precision oncology, demonstrating the potential of genomic-guided treatment selection to advance survival outcomes and improve therapeutic efficacy in lung cancer management.

## Figures and Tables

**Table 1:** Demographics of Combined Training and Testing Datasets.

	Training Set (*n* = 124)	Testing Set (*n* = 31)
**Treatment Received**		
Adjuvant Chemotherapy (ACT)	50	13
Observation (OBS)	74	18
**Age**		
Less than 65	49	17
Older than or equal to 65	75	14
**Stage of Disease**		
I	74	15
II	25	8
III	24	8
IV	1	0

**Table 2:** Random Survival Forest Variable Importance Score.

Variable	Variable Importance Score	Gene Symbol	Gene Descriptions from SynGo Consortium [38]
209660_at	0.008556	TTR	Transthyretin [39]
212215_at	0.007373	PREPL	prolyl endopeptidase like [40]
227000_at	0.007349	MTURN	maturin, neural progenitor differentiation regulator homolog [41]
227200_at	0.006915	ETV3	ETS variant transcription factor 3 [42]
218811_at	0.006703	ORAI2	ORAI calcium release-activated calcium modulator 2 [43]
228886_at	0.006037	LRRC27	leucine-rich repeat containing 27 [44]
240184_at	0.006008	SYNPR-AS1	SYNPR antisense RNA 1 [45]
218230_at	0.005832	ARFIP1	ADP ribosylation factor interacting protein 1 [46]
225012_at	0.005657	HDLBP	high-density lipoprotein binding protein [47]
205504_at	0.005625	BTK	Bruton tyrosine kinase [48]
208683_at	0.005619	CAPN2	calpain 2 [49]
203126_at	0.005215	IMPA2	inositol monophosphatase 2 [50]
225273_at	0.005053	WWC3	WWC family member 3 [51]
207249_s_at	0.004782	SLC28A2	solute carrier family 28 member 2 [52]
206211_at	0.004512	SELE	selectin E [53]
229145_at	0.004417	ANAPC16	anaphase promoting complex subunit 16 [54]
226146_at	0.004407	HEIH	hepatocellular carcinoma up-regulated EZH2-associated long non-coding RNA [55]
235352_at	0.004392	MR1	major histocompatibility complex, class I-related [56]
234297_at	0.004382	RGS8 and SDHAP3	regulator of G protein signaling 8 and SDHA pseudogene 3 [57]
224650_at	0.004321	MAL2	mal, T cell differentiation protein 2 [58]
218693_at	0.004226	TSPAN15	tetraspanin 15 [59]
218707_at	0.004064	ZNF444	zinc finger protein 444 [60]
233167_at	0.003896	SELENOO	selenoprotein O [61]
209682_at	0.003893	CBLB	Cbl proto-oncogene B [62]
200667_at	0.003872	UBE2D3	ubiquitin-conjugating enzyme E2 D3 [63]
229970_at	0.003856	KBTBD7	kelch repeat and BTB domain containing 7 [64]
219468_s_at	0.003791	CUEDC1	CUE domain containing 1 [65]
205448_s_at	0.003735	MAP3K12	mitogen-activated protein kinase kinase kinase 12 [66]
201236_s_at	0.003712	BTG2	BTG anti-proliferation factor 2 [67]
214623_at	0.003702	FBXW4P1	F-box and WD repeat domain containing 4 pseudogene 1 [68]
221861_at	0.003697	ARRB1	arrestin beta 1 [69]
241208_at	0.003691	PDLIM5	PDZ and LIM domain 5 [70]

**Table 3:** Comparison of Survival Models for ACT Recommendation in NSCLC.

Model	Assumptions	Handles Nonlinearity/Interactions	Training C-Index	Test C-Index	Survival Curve Separation	Interpretability	Notable Strengths
Bagging Cox (Elastic Net)	Proportional hazards, linear effects	Limited (via penalization only)	0.996	0.709	Moderate	High	Simple, interpretable, stable with bagging
Random Survival Forest (RSF)	Nonparametric	Yes	0.889	0.885	Strong	Moderate	Best test performance, good at capturing interactions
DeepSurv Neural Network	Flexible, neural Cox model	Yes (deep architecture)	0.990	0.982	Weak to moderate	Low	High predictive accuracy, handles complex relationships

## Data Availability

The datasets analyzed during the current study (GSE37745 and GSE29013) are publicly available from the NCBI Gene Expression Omnibus (GEO). Due to file size and access logistics, we have not provided direct download links; however, the data can be made available upon reasonable request from the first or second author.
